# Restoring failed inhibition in the substantia nigra pars reticulata suppresses absence seizures in rats

**DOI:** 10.1111/epi.18701

**Published:** 2025-11-03

**Authors:** Devin Palmer, Patrick A. Forcelli

**Affiliations:** ^1^ Department of Pharmacology & Physiology Georgetown University Washington DC USA; ^2^ Interdisciplinary Program in Neuroscience Georgetown University Washington DC USA; ^3^ Department of Neuroscience Georgetown University Washington DC USA

**Keywords:** absence epilepsy, deep brain stimulation, optogenetics, spike‐and‐wave discharge

## Abstract

**Objective:**

For over four decades, the substantia nigra pars reticulata (SNr) has been recognized as a critical structure in the modulation of seizure activity. Pharmacological and optogenetic inhibition of the SNr produces robust seizure suppression in a range of seizure models. These findings have given rise to a longstanding, yet unresolved question: do seizures involve a failure of inhibition within the SNr?

**Methods:**

We recorded single‐unit activity in the SNr during spike‐and‐wave discharges (SWDs) in male and female WAG/Rij rats, a model of genetic absence epilepsy. We monitored extracellular γ‐aminobutyric acid (GABA) levels using intensity‐based GABA sensing fluorescence reporter (iGABASnFR). To emphasize the multi‐modal efficacy of SNr inhibition on seizure suppression, we optogenetically inhibited the SNr.

**Results:**

Fifty percent of recorded neurons exhibited a marked increase in firing at SWD onset, with activity returning to baseline at SWD termination. Extracellular GABA levels revealed a decrease in fluorescence during SWDs, consistent with reduced GABAergic transmission. Optogenetic inhibition of SNr neurons using continuous (open‐loop) inhibition, but not closed‐loop (responsive) inhibition, significantly reduced SWD incidence.

**Significance:**

These data suggest that a loss of GABAergic input to the SNr is associated with increased neuronal activity. Optogenetically restoring inhibition effectively reduced seizure burden. Together, these findings address a long‐standing gap in the literature and provide compelling evidence that impaired inhibition within the SNr contributes to seizure expression.

**Key points**

- Neurons in the substantia nigra pars reticulata (SNr) display a marked increase in firing at the onset of spike‐and‐wave discharges (SWDs).
- Extracellular γ‐aminobutyric acid (GABA) levels fall during SWDs.
- Opotogenetic inhibition of the SNr suppresses seizures in WAG/Rij rats.

## 1 INTRODUCTION

For over four decades, the substantia nigra pars reticulata (SNr) has been recognized as a critical structure in the modulation of seizure activity.^1^ Inhibition of the SNr and disinhibition of SNr projection targets suppresses seizures. For example, focal infusion of γ‐aminobutyric acid (GABA) receptor agonists or inhibitors of GABA breakdown into the SNr suppress seizures, suggesting that elevating GABA‐mediated neurotransmission within the SNr can be exploited as an anti‐seizure therapy.^2^, ^3^ Similarly, lesions and optogenetic inhibition of the SNr potently suppress seizure activity in a wide range of seizure models.^2^, ^4^, ^5^, ^6^ This anti‐seizure effect is mediated in part through disinhibition of the superior colliculus.^5^, ^7^, ^8^ Consistent with this, pharmacological and optogenetic activation of the superior colliculus suppresses seizures in several models.^5^, ^9^, ^10^

Just as inhibiting the SNr *decreases* seizures, impairing inhibition within the SNr (e.g., through inhibition of GABA synthesis or blockade of GABA receptors) *increases sensitivity* to pharmacologically or electrically evoked seizures, raising the possibility that failure of inhibition within this region results in a *pro‐seizure* state.^11^, ^12^ Consistent with this, microdialysis studies in the genetically epilepsy prone rat (GEPR) revealed impaired GABA release in the SNr.^13^

This system, which has been proposed to exert endogenous control over seizures has been referred to as the “nigral inhibitory system” and provides a remote “choke point” on seizure activity.^14^ Although the extended basal ganglia circuitry is not critical for seizure initiation, it has potent effects on brain‐wide synchronization^15^ – for example through descending control of the pedunculopontine nucleus and reticular activating system.

SNr neurons are regular spiking GABAergic neurons with a high tonic firing rate. They provide tonic inhibitory control over basal ganglia projection targets such as the thalamus, superior colliculus, and pedunculopontine nucleus. Firing of SNr neurons is regulated by input from the striatal direct and indirect pathways.^16^ Striatal direct pathway neurons, which synapse directly onto SNr output cells, are also GABAergic, and thus, activity within the direct pathway suppresses SNr firing. By contrast, activity in the indirect pathway elevates SNr firing via disinhibition of glutamatergic input from the subthalamic nucleus. These pathways thus act as a “break” and an “accelerator,” respectively, on nigral firing.^16^ A central hypothesis regarding the basal ganglia's role in the control of seizures is that circuit manipulations that *decrease* firing in the SNr will result in *anti‐seizure* effects.

Only a small number of studies have recorded the activity of SNr neurons during or after seizures, and they have been limited by small sample sizes, and were conducted predominantly in models of acutely evoked seizures. Accordingly, results from these studies have been mixed, with some demonstrating increased SNr firing during seizures, others demonstrating no change, and others indicating regional variability.^17^, ^18^, ^19^, ^20^, ^21^ This leaves a long‐standing question still unresolved: does spontaneous seizure generation involve a failure of inhibitory control within the SNpr?

To address this, we turned to the WAG/Rij rat model. The WAG/Rij rat is an inbred rat strain displaying SWDs (7–8 Hz), phenotypically similar to clinical absence seizures.^22^ We recently reported that single unit activity in the deep and intermediate layers of the superior colliculus (DLSC), one of the primary projection targets of the SNr, is suppressed in the seconds leading up to the start of SWDs.^23^ We have previously demonstrated (in a pharmacologically‐evoked absence seizure model) that inhibition of the SNr suppresses spike‐and‐wave discharges.^5^ Here, through a combination of single unit neurophysiology, fiber photometry, and optogenetics we demonstrate that: (1) neurons in the SNr display a marked increase in firing at the onset of SWDs, (2) that extracellular GABA levels fall during SWDs, and (3) that optogenetic inhibition of the SNr suppresses seizures in WAG/Rij rats.

## 2 MATERIALS AND METHODS

### 2.1 Animals

Adult (4‐ to 6‐month‐old) female and male WAG/Rij rats from the Georgetown University colony were used (crosses from a breeding pair obtained from Charles River Laboratories, Italy). Two animals were housed per cage in a temperature and humidity‐controlled room in the Division of Comparative Medicine (Georgetown University) with food (Lab Diet #5001) and water ad libitum. All experiments were performed during the light phase of the light/dark cycle (6:00 am to 6:00 pm). Procedures were approved by the Georgetown Institutional Care and Use Committee (#2016–1184) and were consistent with the Guide for the Care and Use of Laboratory Animals and ARRIVE (Animal Research: Reporting of In Vivo Experiments) Guidelines. A total of 53 animals were used in these experiments.

Animals were used in three experiments: (1) in vivo single‐unit electrophysiology recordings, (2) fiber photometry to measure GABA levels, and (3) optogenetic modulation of SNr activity. For electrophysiology experiments, 11 male and 9 female rats were implanted with a 16‐channel microwire array in the SNr. For photometry experiments, 3 male and 2 female rats were injected bilaterally (*n* = 2 per animal) with pAAV1‐iGABASnFR‐WPRE‐SV40 and a fiber was implanted above the injection site. iGABASnFR is a validated approach for in vivo measurement of GABA dynamics and shows a high degree of specificity for GABA.^24^ Thirteen male and 16 female rats were used for optogenetic experiments using the same coordinates and procedures we have used previously for optogenetic inhibition of the SNr.^5^ Animals were randomized into either the inhibitory virus (Archaerhodopsin; ArchT) or control (Green fluorescent protein; GFP) condition; 1 male and 1 female were excluded based on incorrect viral targeting. (See Appendix S1 for surgical methods.)

### 2.2 Single unit recording and analysis

Data were collected during a 2 h session using an OpenEphys acquisition system, as we have described previously.^23^ (See Appendix S1 for additional details on data acquisition and processing.) We generated peri‐event time histograms for each unit, time locking the activity to either the start or end of SWDs. Activity patterns were classified based on firing rates relative to the baseline activity for the unit. We classified activity patterns in four time windows: Before SWD Start (i.e., 2.5 s preceding an SWD), After SWD Start (i.e., 2.5 s following the first spike in an SWD), Before SWD End (i.e., 2.5 s prior to the last spike in an SWD), and After SWD End (i.e., 2.5 s after the last spike in an SWD).

Units with activity that fell below the 99% confidence intervals (CIs) for baseline firing were classified as “phasic decrease.” Units with activity that were above the 99% CIs for baseline firing were classified as “phasic increase.” Units that did not exceed either CI were classified as tonic. Units were categorized as either putative GABAergic or putative dopaminergic cells based on waveform shape.^25^ Details of the analysis of phase‐locking are presented in the Appendix S1.

### 2.3 Fiber‐photometry recording and analysis

Acquisition was performed with a Neurophotometrics fiber photometry system (FP3001, Neurophotometrics LTD). The photometry signal was processed using a custom MatLab script. Acquisition, preprocessing, and processing are described in the Appendix S1. Processed data (∆F/F) were time locked to the electroencephalography (EEG signal. PreSWD, SWD, and PostSWD variables were generated as described. z‐score ∆F/F were generated from the 30 s prior to SWD start through 30 s after SWD. Area under the curve (AUC) values were calculated after SWD start and end and analyzed to baseline levels.

### 2.4 Optogenetic neuromodulation and EEG recording

EEG recording sessions were performed as described in Appendix S1. Light was delivered at 100 Hz with 50% duty cycle with a 5 ms pulse width. For open‐loop modulation, light pulses (green light, λ = 560 nm) were delivered for the duration of the observation period (120 min) to both inhibitory vector (opsin positive) and control vector (opsin negative) animals. The frequency of light delivery was based on our prior study inhibiting the SNr in an acute absence seizure model.^5^ We quantified the number and duration of each SWD during the 2‐h long observation period. (See Appendix S1 for closed‐loop neuromodulation procedures.)

### 2.5 Statistics

Statistical analyses were performed using GraphPad Prism and Matlab. Firing rates were analyzed by one‐ or two‐way analysis of variance (ANOVA). Within‐unit changes in firing rate were analyzed by paired *t* tests. Optogenetic inhibition dependent measures (total seizure duration, number of SWDs, average SWD duration) were analyzed by paired *t* tests (comparing no light delivery to light delivery). The proportion of cells displaying each activity pattern was analyzed by permutation testing.

## 3 RESULTS

### 3.1 Histological confirmation

**FIGURE 1:**
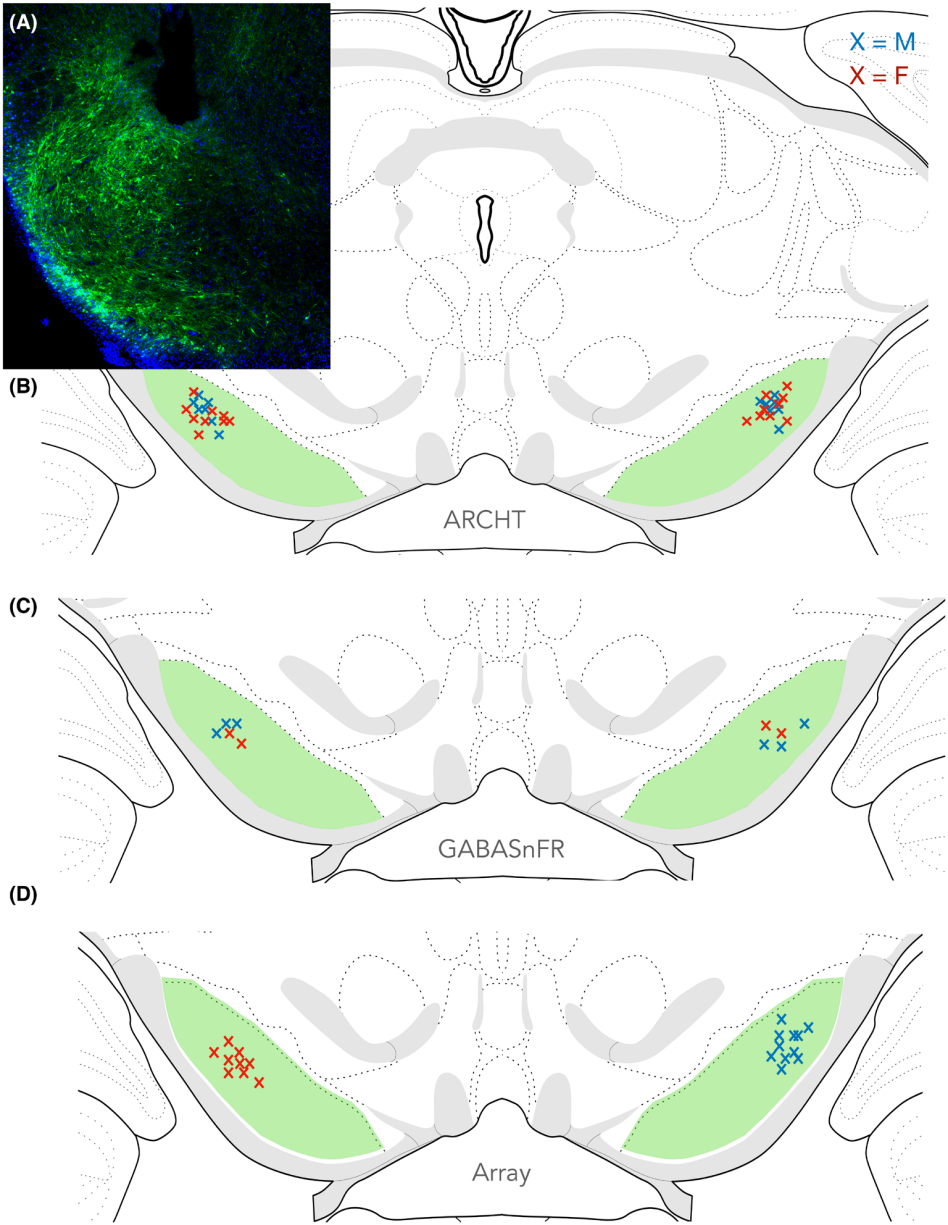
Histological verification of virus (ArchT, iGABASnFr) expression and optics and array location in the substantia nigra pars reticulata (SNr). (A) Coronal section through the SNr. Green indicates GFP fluorescence from the injected virus. (B) Optic placement for optogenetic studies for male and female ArchT animals (C), optic placement for fiber‐photometry studies for male and female iGABASnFR animals, and (D) array placement for in vivo electrophysiology studies for male and female animals. Male (blue x), female (red x).

Microinjection of ArchT‐GFP (Figure 1A) robustly labeled the SNr, as we have described previously.^5^ Viral expression for optogenetic and photometry experiments was ventral to the cannula tip in both hemispheres. As seen in Figure 1B–D, surgical implantation of fiber optics and array electrodes were placed in the dorsolateral SNr. Electrodes and fiber optics were confined to a narrow rostrocaudal range (centered on AP‐5.5) as intended.

### 3.2 Single‐unit activity of the SNr during SWDs

**FIGURE 2:**
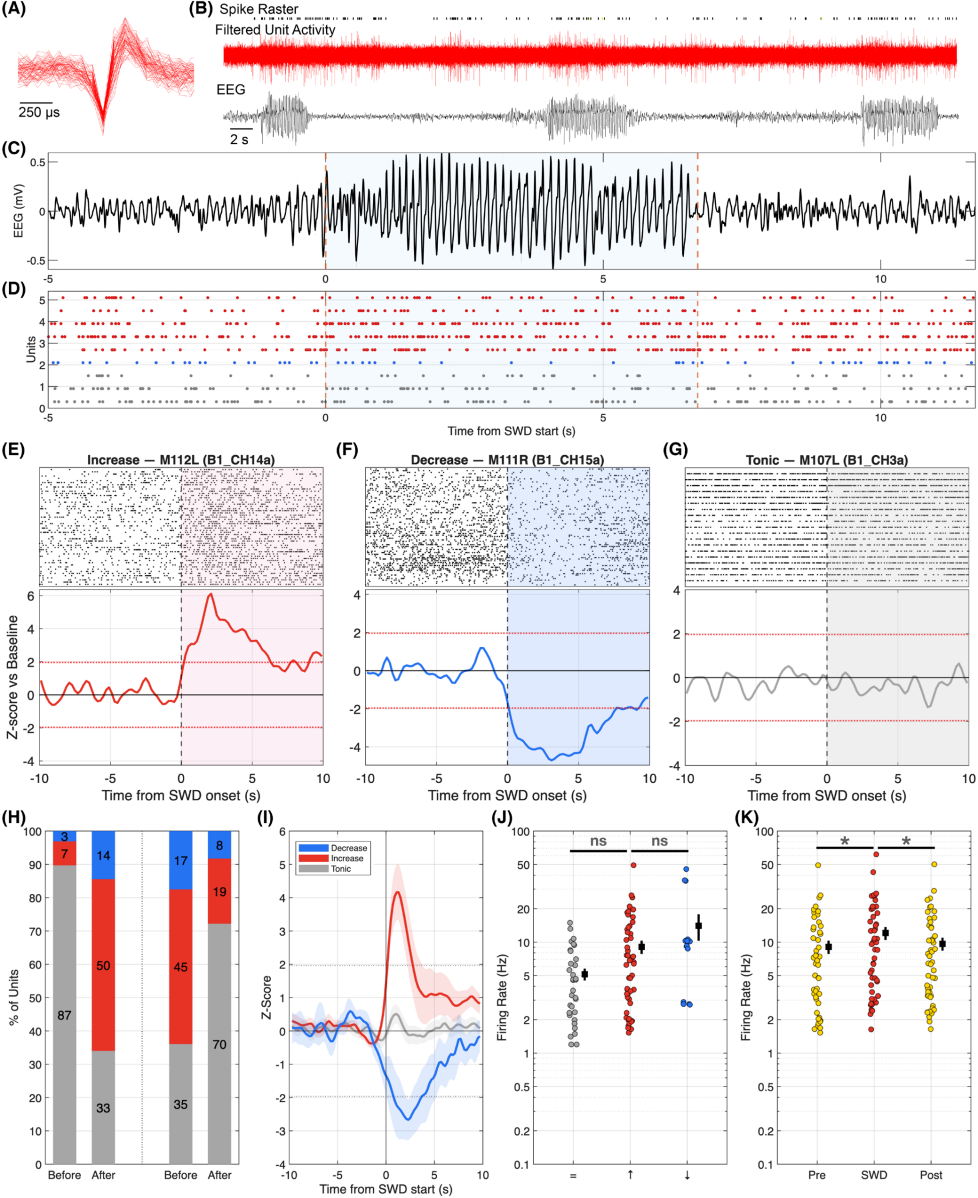
Single‐unit GABAergic activity within the substanita nigra pars reticulata (SNr) increases after spike‐and‐wave discharge (SWD), D onset. (A) Representative waveform from a putative GABAergic unit. (B) Raster plot (top) of a single unit as it correlates to the raw spiking activity of a single channel (middle, red) and SWD activity (bottom). (C) Expanded view of a single SWD time locked to (D) raster plots for a phasic increase (red raster), phasic decrease (blue raster), and tonic unit (gray raster). (E) Raster plot (top) matched to the peri‐event histogram of a unit showing tonic, (F) phasic increase and (G) phasic decrease preceding seizure start (seizure start = 0). Gray‐shaded region indicates tonic, pink‐shaded region indicates increase, and blue‐shaded region indicates decrease after SWD onset. (H) Percentage of units in each response category for each time bin. Phasic increases in firing were defined as firing rates that were above the 95% confidence intervals (CIs) for the baseline firing rate of the unit in the after SWD start. Baseline firing rates were defined as the average activity for the unit for the duration of the recording excluding PreSWD, SWD, and PostSWD time periods. In the before SWD start bin, responses did not differ from chance. In the after SWD start and before SWD end bins, the number of “up” and “down” units were both elevated relative to chance (evaluated by permutation tests). (I) Average activity in units that showed increased firing at SWD onset showed sustained increase through SWD; similar sustained activity was seen in both decreased and tonic units. Tonic units displayed no significant changes in firing rate. (Line indicates mean; shaded areas show the 95% CIs). (J) Baseline firing rates did not differ between unit profiles (ANOVA, *F*_2,89_ = 1.598, *p* = .2080, *n* = 14 “decrease”; *n* = 50 “increase; *n* = 33 “tonic”). (K) For units that increased in activity a significant effect of time bin (ANOVA, Time: *F*_1.796,88.03_ = 65.79, *p* < .0001): Firing rates were higher during the SWD periods compared with the PreSWD and PostSWD periods; *p* < .0001 and *p* < .0001, respectively. *p < .05, **p < .01, ***p < .001, ****p < .001.

We recorded from 110 well‐isolated units in the SNr of WAG/Rij rats, with activity time locked to a mean of 28 SWDs per array. Units were categorized as either GABAergic (Figure 2A) or dopaminergic (Figure S1A) based on waveform shape and half width (Figure S1B). Figure 2B shows a unit raster in relation to the filtered channel spike activity (middle trace) and SWDs (bottom trace) for a putative GABAergic neuron.

For each unit, we next analyzed activity time‐locked to the start and end of spike SWDs (Figure 2C). We categorized activity patterns at the start of SWDs as either phasic increase (Figure 2D – red raster, Figure 2E), phasic decrease (Figure 2D – blue raster, Figure 2F), or tonic (no change from baseline firing, Figure 2D – gray raster, Figure 2G) based on their z‐scored firing rates relative to the pre‐SWD period. We analyzed the proportion of units displaying each response type in the 5 s before or 5 s following the start or end of an SWD. For each unit, we calculated the baseline probability of an increase, decrease, or tonic response by resampling non‐SWD segments of the recordings, and evaluated the observed vs resampled rates by permutation testing. A greater‐than‐chance (*p* < .001) proportion of units displayed a phasic increase in activity after SWD (Figure 2H), whereas a smaller absolute number of units displayed a decrease in activity after SWD start; this was also highly significant (*p* < .00001, permutation test). Although our aim was to record from GABAergic neurons in the SNr, by chance we recorded from a small number of dopaminergic (DA) units. These units had increased responses similar to SWDs (Figure S1G).

Z‐transformed firing rates of tonic, phasic decreasing and phasic increasing units at the start of the SWD are depicted in Figure 2I. Baseline firing rate did not differ across the activation profiles (Figure 2J). For units that displayed a phasic increase in firing after SWD start, firing rate was elevated both relative to the pre‐ and post‐SWD periods (Figure 2K).

**FIGURE 3:**
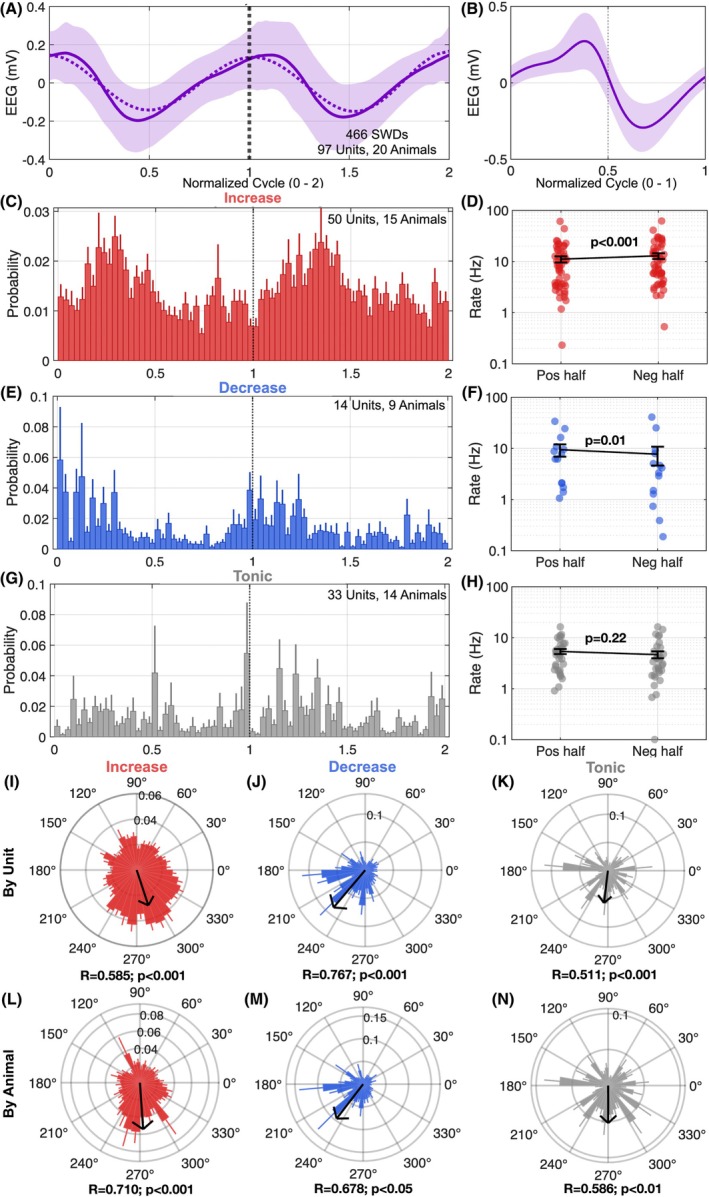
SWD phase preference for GABA unit categories. (A) Normalized first two cycles of SWDs across a total of 466 SWDs. (B) Representative SWD Cycle 1 for a single animal. (C) Firing probability of all phasic increase GABA units within the first two cycles of the SWD (200 ms time‐bins) showed (D) increased firing rates in the negative half of the SWD phase (Wilcoxon signed‐rank test, *W* = 647, *p* = .001). (E) Firing probability of all phasic decrease GABA units within the first two cycles of the SWD showed (F) increased firing rates in the positive half of the SWD phase (Wilcoxon signed‐rank test, *W* = −77, *p* = .0134). (G) Firing probability of all tonic GABA units within the first two cycles of the SWD showed (H) no phase specific differences in firing rate (Mann–Whitney *U* = 449.5, *p =* .2260). A Rayleigh test revealed a significant departure from uniformity by unit for (I) phasic increase units (*z*(50) = 17.13, mean angle = 288.3, *p <* .001), (J) phasic decrease (*z*(14) = 8.24, mean angle = 229.7, *p* < .001), and (K) tonic units (*z*(31) = 8.11, mean angle = 263.2, *p* < .001). Polar plots showing phase‐specific firing rate probability by animal for (L) phasic increase (*z*(15) = 7.57, mean angle = 273.9, *p <* .001), (J) phasic decrease (*z*(9) = 4.13, mean angle = 232.5, *p* = .011), and (K) tonic units (*z*(13) = 4.46, mean angle = 270.1, *p* = .009).

We next evaluated the firing rate probability of all GABA units in relation to SWD phase (Figure 3A,B). Units that displayed an increase in firing after SWD onset also displayed significant phase locking, with higher firing rates during the negative phase of the EEG spike (Figure 3C,D). By contrast, units that decreased firing after SWD start were more likely to fire in the positive half of SWDs (Figure 3E,F). Tonic units did not show any significant phase preference (Figure 3G,H). Polar plots of SWD phase preference for each activity profile were consistent and significant for both increase and decrease unit types when analyzed at the level of unit (Figure 3I–K) or when units were averaged within animal (Figure 3L–N). We repeated this analysis for only the first spike in each SWD, as well as for all spikes in all SWDs, and found similar responses (Figure S2).

### 3.3 Afferent GABA release decreases in SNr during SWDs

**FIGURE 4:**
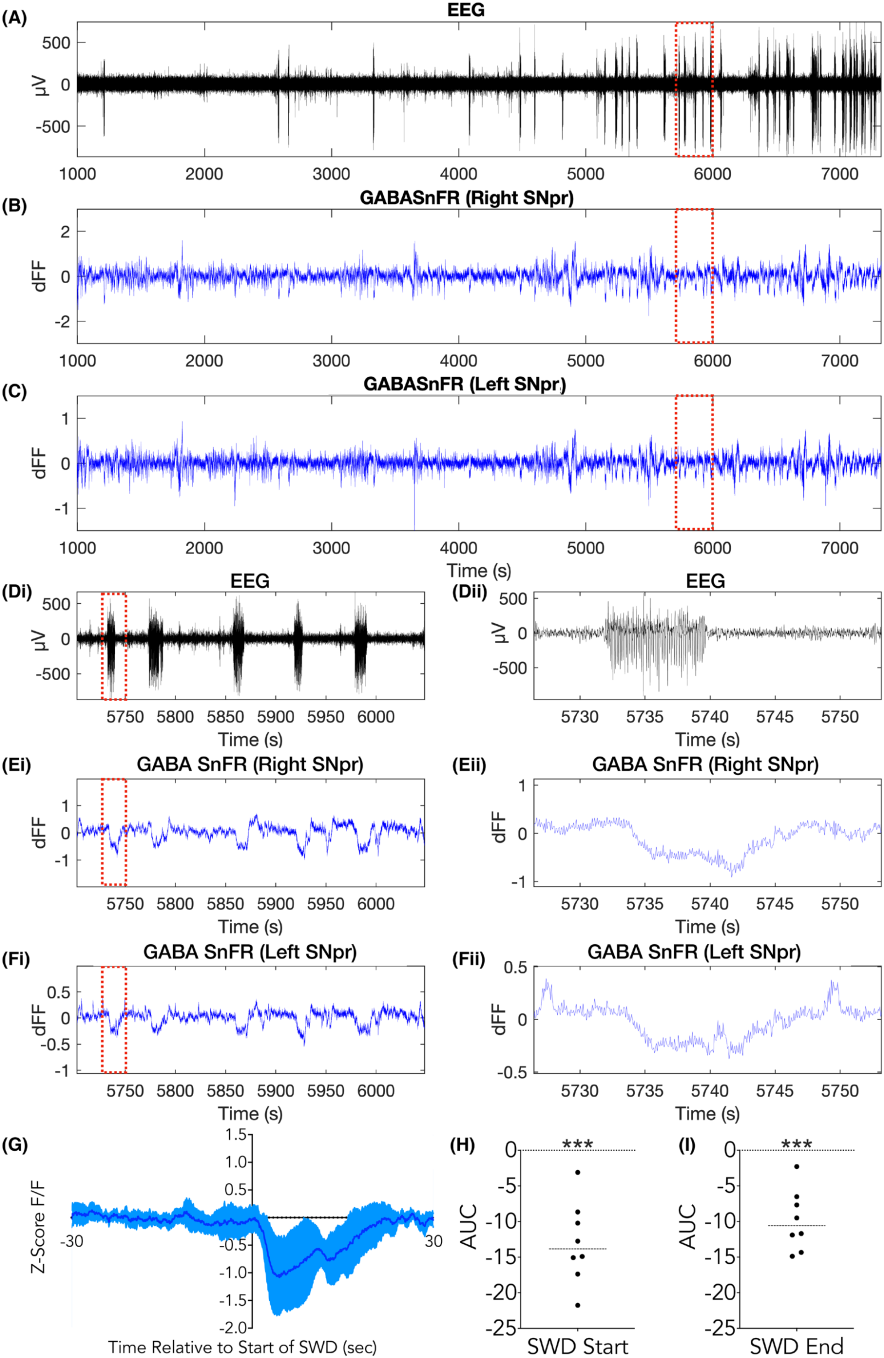
Decrease in afferent GABA within the substantia nigra pars reticulata (SNr) during spike‐and‐wave discharges (SWDs). (A) Raw electroencephalogram (EEG) signal from a representative WAG/Rij rat recorded over a 2 h session. (B) Normalized change in fluorescence (dFF) signal from the right and (C) left hemispheres of the same animal and recording session as in A. (Di, Dii) Data from panelA shown at expanded timescales (5 min and 30 s). (Ei, Eii) Data from B shown at expanded timescales (5 min and 30 s), showing decreases in GABASnFr dFF time‐locked to SWDs in Di. (Fi, Fii) Expanded timescale of the dFF signal shown in C. (G) Average z‐score ∆*F*/*F* during SWDs across all animals and hemispheres. (H) Area under the curve (AUC) values for the iGABASnFR signal at SWD start were significantly different from baseline levels (paired *t* test, *t* = 6.43, df = 7 *p* = .0004). (I) AUC values for the iGABASnfR at SWD end were significantly different from baseline levels (paired *t* test, *t* = 6.56, df = 7, *p* = .0003). ***p < .001.

The primary source of inhibitory control over SNr neurons is GABAergic input from the striatum. Elevating GABA in the SNr is potently anticonvulsant,^2^ and similarly, depleting GABA within the SNr is proconvulsant.^26^ However, no study has demonstrated real‐time modulation of GABA in the SNr during seizures. To address this gap, and to determine if increased firing in the SNr was associated with decreased GABA release, we recorded, bilaterally, from four WAG/Rij rats (*n* = 8 hemispheres) injected with a fluorescent GABA sensor (iGABASnFR).^24^ SWDs (Figure 4, Di–Dii) were time locked to the fluorescent signal (Figure 4, C,Ei–Fii) for each animal. Across all animals, average z‐scored ∆F/F values illustrate a marked decrease in iGABASnFR fluorescence during SWDs (Figure 4G). We calculated the AUC values for each animal and hemisphere at SWD start and prior to SWD end, and we compared each to a set AUC of 0 (representing no change). GABA levels were decreased robustly in the SNr during SWDs (Figure 4H,I). In sum, afferent GABA release within the SNr decreased during SWDs, and the timing of this decrease is consistent with the increased firing we observed in our single unit experiments.

### 3.4 Optogenetic inhibition of the SNr

**FIGURE 5:**
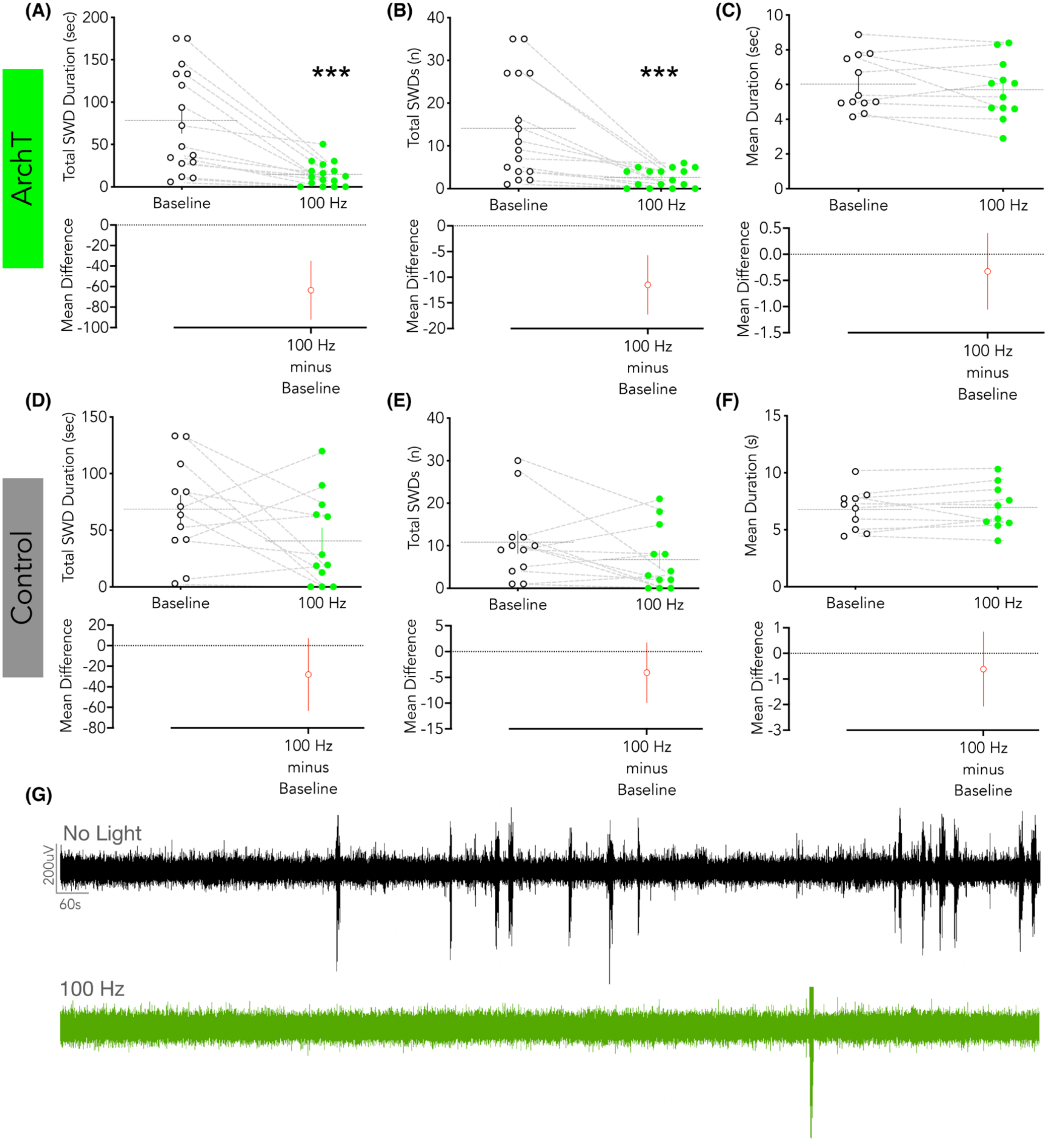
Open‐loop optogenetic inhibition of the SNr suppresses absence‐like seizures in WAG/Rij rats. (A) Total seizure duration (paired *t* test, *t* = 4.763, *p* = .0003) and (B) number of SWDs were reduced significantly by 100 Hz light delivery in ArchT‐expressing WAG/Rij rats paired *t* test, (paired *t* test, *t* = 4.269, *p* = .0007), whereas (C) mean SWD duration was unchanged (paired *t* test, *t* = .9932, *p* = .3420). Note that several animals were excluded from the analysis of average SWD duration, as they displayed no SWDs during the 30 min session (excluded: 100 Hz: *N* = 4) and this would have artificially reduced the SWD duration. In control vector rats, no measure of SWDs was altered by the light delivery: (D) total duration (paired *t* test, *t* = 1.752, *p* = .1076), (E) number (paired *t*‐test, *t* = 1.549, *p* = .1496), and (F) mean duration (paired *t* test, *t* = .9579, *p* = .3631). Plots show individual replicates with mean and standard error. Mean differences (treated – control, with 95% confidence intervals) are plotted below each variable. (G) Representative EEG traces under no light delivery and 100 Hz light delivery conditions in ArchT‐expressing (top) and control‐vector rats (bottom). ***p < .001.

Given that we observed both an increase in SNr firing and a failure of GABAergic inhibition within the SNr in association with SWDs, we next inhibited SNr neuron activity. On a within‐subject basis, we compared open‐loop (continuous) 100 Hz optogenetic inhibition of the SNr. As shown in Figure 5, inhibition reduced the total SWD duration (Figure 5A) and the number of SWDs (Figure 5B). The average SWD duration was unchanged as a function of light delivery (Figure 5C). By contrast, in opsin‐negative control animals, light delivery had no effect on any parameter (Figure 5D–F). Total seizure duration, number of SWDs, and average SWD duration were unchanged as a function of light delivery. By contrast, closed‐loop 100 Hz optogenetic inhibition of the SNr showed no effect on SWDs in either opsin‐expressing or opsin‐negative control rats (Figure S2A,B).

## 4 DISCUSSION

Although prior studies have demonstrated robust anti‐seizure effects of inhibiting the SNr in rodents and non‐human primates,^2^, ^3^, ^4^, ^5^, ^6^ evidence for seizure‐associated changes in activity within the SNr have produced conflicting results. Studies in kindled rats, in particular, have yielded mixed results ranging from no changes in baseline firing rate after kindling^18^ to elevated firing in the posterior, but not anterior SNr a day after a seizure,^19^ to correlated firing between the amygdala and SNr neurons during seizures.^20^ Focal motor seizures evoked by cortical penicillin injection in macaques likewise found no alteration in SNr firing rate.^21^ Of interest, several reports have demonstrated a suppression of single unit activity within the SNr after systemic administration of anti‐seizure medications.^27^, ^28^ Only one prior study examined SNr single unit activity during generalized seizures, albeit with a small sample size (*n* = 20 units). Deransart and colleagues reported elevated SNr firing rates during absence‐like seizures in the GAERS rat model.^17^ Consistent with this, here we report elevated firing rates of GABA neurons within the SNr during spontaneous absence‐like seizures in the WAG/Rij rat model. Whether the SNr is differentially modulated in focal as compared to generalized seizures, or rather, whether absence‐like seizures, as compared to those originating in other circuits, explain the heterogeneity in findings remains to be determined.

GABAergic neurons in the SNr fire at a relatively high tonic rate; their activity is controlled by excitatory input from the subthalamic nucleus and inhibitory input from the striatum. Given the striking anti‐seizure effects reported following focal application of GABA agonists to the SNr, we hypothesized that seizures would be associated with transient decreases in GABA levels. We found that consistently, both within and between subjects, that GABA levels as measured using iGABASnfR fluorescence in the SNr were reduced during SWDs in WAG/Rij rats. These data suggest that a failure of striatonigral inhibition co‐occurs with SWDs, which in turn may explain the elevated single unit activity we observed. Consistent with this, activation of striatal projection neurons, which is predicted to increase efferent GABA release in the SNr, suppresses seizures, including absence‐like seizures.^29^, ^30^, ^31^ Moreover, a recent functional magnetic resonance imaging (fMRI) and single‐unit ensemble recording study in the Genetic Absence Epilepsy Rat from Strasbourg (GAERS) model found decreased blood oxygen level (BOLD) signal in the lateral caudate coincident with SWD onset.^32^

We found that open‐loop optogenetic inhibition of the SNr reduced seizure burden in WAG/Rij rats. This is consistent with prior pharmacological^2^ and optogenetic^5^ studies in acutely evoked seizure models, and extends these findings to spontaneous seizures. We have previously reported that selective silencing of projections from the SNr to DLSC suppresses seizures,^5^ and that both closed‐loop and open‐loop activation of the DLSC robustly suppresses seizures in the WAG/Rij rat.^23^ Of interest, here we found that although open‐loop inhibition of the SNr suppressed SWDs, closed‐loop inhibition did not. This may reflect a relatively brief period of enhanced excitation within the SNr during SWDs; as our closed‐loop manipulations lag ~1–2 s from the onset of a seizure, we may be “missing” the optimal window for intervention.

Despite decades of interest, the SNr has not been well examined as a clinical target for deep brain stimulation. In fact, only a single small case series and one single case report have evaluated the impact of SNr deep brain stimulation in epilepsy.^33^, ^34^ Our data demonstrate that the anti‐seizure effects of SNr manipulations extend beyond acutely evoked seizures, to chronic, spontaneous seizures. Although the present work focused on absence seizures, future studies should examine other models of spontaneous seizures. Together this work further strengthens the case for clinical examination of this region as a deep brain stimulation target.

## 5 CONCLUSION

Here we found that activity of single units in the SNr increases in response to seizures in the WAG/Rij model of absence epilepsy, a response likely driven by a decrease in afferent GABA release within the SNr. Reducing SNr activity through continuous (open‐loop) optogenetic inhibition potently reduces seizure burden while closed‐loop responsive optogenetic inhibition does not. These data provide the first evidence for a real‐time failure of nigral inhibition in association with seizures, addressing a long‐standing question in the field, and further indicating the potential for SNr‐based brain stimulation to control seizure.

## AUTHOR CONTRIBUTIONS

D.P. and P.A.F. designed experiments, D.P. conducted experiments, D.P. and P.A.F. analyzed data, P.A.F. wrote code, and D.P. and P.A.F. wrote the manuscript.

## CONFLICT OF INTEREST STATEMENT

None of the authors have any conflicts of interest to disclose. We confirm that we have read the Journal's position on issues involved in ethical publication and affirm that this report is consistent with those guidelines.

## Supporting information


Appendix S1.

## Data Availability

The data that support the findings of this study are iavailable via Dryad (DOI: https://doi.org/10.5061/dryad.66t1g1kds).

## References

[1] Hikosaka O , Wurtz RH . Visual and oculomotor functions of monkey substantia nigra pars reticulata. IV. Relation of substantia nigra to superior colliculus. J Neurophysiol. 1983;49(5):1285–1301. 6306173 10.1152/jn.1983.49.5.1285

[2] Iadarola MJ , Gale K . Substantia nigra: site of anticonvulsant activity mediated by gamma‐aminobutyric acid. Science. 1982;218(4578):1237–1240. 10.1126/science.7146907 7146907

[3] Gale K , Dybdal D , Wicker E , Campos‐Rodriguez C , Maior RS , Elorette C , et al. Piriform cortex is an ictogenic trigger zone in the primate brain. Epilepsia. 2024;5:569–582. 10.1111/epi.18201 PMC11929115 39636294

[4] Fan XD , Li XM , Juorio AV . Substantia nigra pars reticulata lesion facilitates kainic acid‐induced seizures. Brain Res. 2000;877(1):107–109. 10.1016/s0006-8993(00)02620-2 10980251

[5] Wicker E , Beck VC , Kulick‐Soper C , Kulick‐Soper CV , Hyder SK , Campos‐Rodriguez C , et al. Descending projections from the substantia nigra pars reticulata differentially control seizures. Proc Natl Acad Sci U S A. 2019;116(52):27084–27094. 10.1073/pnas.1908176117 31843937 PMC6936676

[6] Deransart C , Depaulis A . The control of seizures by the basal ganglia? A review of experimental data. Epileptic Disord. 2002;4(Suppl 3):S61–S72. 12495876

[7] Redgrave P , Simkins M , overton P , Dean P . Anticonvulsant role of nigrotectal projection in the maximal electroshock model of epilepsy–I. Mapping of dorsal midbrain with bicuculline. Neuroscience. 1992;46(2):379–390. 10.1016/0306-4522(92)90059-b 1542413

[8] Soper C , Wicker E , Kulick CV , N'Gouemo P , Forcelli PA . Optogenetic activation of superior colliculus neurons suppresses seizures originating in diverse brain networks. Neurobiol Dis. 2016;87:102–115. 10.1016/j.nbd.2015.12.012 26721319 PMC4724547

[9] Dean P , Gale K . Anticonvulsant action of GABA receptor blockade in the nigrotectal target region. Brain Res. 1989;477(1):391–395. 10.1016/0006-8993(89)91434-0 2539235

[10] Hyder SK , Ghosh A , Forcelli PA . Optogenetic activation of the superior colliculus attenuates spontaneous seizures in the pilocarpine model of temporal lobe epilepsy. Epilepsia. 2023;64(2):524–535. 10.1111/epi.17469 36448878 PMC10907897

[11] Maggio R , Sohn E , Gale K . Lack of proconvulsant action of GABA depletion in substantia nigra in several seizure models. Brain Res. 1991;547(1):1–6. 10.1016/0006-8993(91)90567-F 1650283

[12] Löscher W , Czuczwar SJ , Jäckel R , Schwarz M . Effect of microinjections of gamma‐vinyl GABA or isoniazid into substantia nigra on the development of amygdala kindling in rats. Exp Neurol. 1987;95(3):622–638. 3817084 10.1016/0014-4886(87)90304-9

[13] Doretto MC , Burger RL , Mishra PK , Garcia‐Cairasco N , Dailey JW , Jobe PC . A microdialysis study of amino acid concentrations in the extracellular fluid of the substantia nigra of freely behaving GEPR‐9s: relationship to seizure predisposition. Epilepsy Res. 1994;17(2):157–165. 10.1016/0920-1211(94)90015-9 8194511

[14] Depaulis A , Vergnes M , Marescaux C . Endogenous control of epilepsy: the nigral inhibitory system. Prog Neurobiol. 1994;42(1):33–52. 7480786 10.1016/0301-0082(94)90020-5

[15] Redgrave P , Dean P . Tonic desynchronisation of cortical electroencephalogram by electrical and chemical stimulation of superior colliculus and surrounding structures in urethane‐anaesthetised rats. Neuroscience. 1985;16(3):659–671. 2869444 10.1016/0306-4522(85)90199-x

[16] Alexander GE , Crutcher MD , DeLong MR . Basal ganglia‐thalamocortical circuits: parallel substrates for motor, oculomotor, “prefrontal” and “limbic” functions. Prog Brain Res. 1990;85:119–146. 2094891

[17] Deransart C , Hellwig B , Heupel‐Reuter M , Léger JF , Heck D , Lücking CH . Single‐unit analysis of substantia nigra pars reticulata neurons in freely behaving rats with genetic absence epilepsy. Epilepsia. 2003;44(12):1513–1520. 10.1111/j.0013-9580.2003.26603.x 14636321

[18] Waszczak BL , Applegate CD , Burchfiel JL . Kindling does not cause persistent changes in firing rates or transmitter sensitivities of substantia nigra pars reticulata neurons. Brain Res. 1988;455(1):115–122. 10.1016/0006-8993(88)90120-5 2901283

[19] Gernert M , Fedrowitz M , Wlaz P , Löscher W . Subregional changes in discharge rate, pattern, and drug sensitivity of putative GABAergic nigral neurons in the kindling model of epilepsy. Eur J Neurosci. 2004;20(9):2377–2386. 10.1111/j.1460-9568.2004.03699.x 15525279

[20] Shi LH , Luo F , Woodward DJ , McIntyre DC , Chang JY . Temporal sequence of ictal discharges propagation in the corticolimbic basal ganglia system during amygdala kindled seizures in freely moving rats. Epilepsy Res. 2007;73(1):85–97. 10.1016/j.eplepsyres.2006.08.008 17049434 PMC1941664

[21] Devergnas A , Piallat B , Prabhu S , Torres N , Louis Benabid A , David O , et al. The subcortical hidden side of focal motor seizures: evidence from micro‐recordings and local field potentials. Brain. 2012;135(7):2263–2276. 10.1093/brain/aws134 22710196

[22] Coenen AM , Van Luijtelaar EL . The WAG/Rij rat model for absence epilepsy: age and sex factors. Epilepsy Res. 1987;1(5):297–301. 10.1016/0920-1211(87)90005-2 3143552

[23] Campos‐Rodriguez C , Palmer D , Forcelli PA . Optogenetic stimulation of the superior colliculus suppresses genetic absence seizures. Brain. 2023;146(10):4320–4335. 10.1093/brain/awad166 37192344 PMC11004938

[24] Marvin JS , Shimoda Y , Magloire V , Leite M , Kawashima T , Jensen TP , et al. A genetically encoded fluorescent sensor for in vivo imaging of GABA. Nat Methods. 2019;16(8):763–770. 10.1038/s41592-019-0471-2 31308547

[25] Fan D , Rossi MA , Yin HH . Mechanisms of action selection and timing in substantia Nigra neurons. J Neurosci. 2012;32(16):5534–5548. 10.1523/JNEUROSCI.5924-11.2012 22514315 PMC6703499

[26] Kalichman MW , Burnham WM , Livingston KE . Pharmacological investigation of gamma‐aminobutyric acid (gaba) and fully‐developed generalized seizures in the amygdala‐kindled rat. Neuropharmacology. 1982;21(2):127–131. 10.1016/0028-3908(82)90151-4 7063109

[27] Waszczak BL , Lee EK , Walters JR . Effects of anticonvulsant drugs on substantia nigra pars reticulata neurons. J Pharmacol Exp Ther. 1986;239(2):606–611. 3095541

[28] Rohlfs A , Rundfeldt C , Koch R , Löscher W . A comparison of the effects of valproate and its major active metabolite E‐2‐en‐valproate on single unit activity of substantia nigra pars reticulata neurons in rats. J Pharmacol Exp Ther. 1996;277(3):1305–1314. 8667191

[29] Hyder SK , Lazarini‐Lopes W , Toib J , Williams G , Sukharev A , Forcelli PA . Optogenetic stimulation of the dorsal striatum bidirectionally controls seizures. Proc Natl Acad Sci U S A. 2025;122(14):e2419178122. 10.1073/pnas.2419178122 40163720 PMC12002315

[30] Cavalheiro EA , Turski L . Intrastriatal N‐methyl‐d‐aspartate prevents amygdala kindled seizures in rats. Brain Res. 1986;377(1):173–176. 10.1016/0006-8993(86)91204-7 3015345

[31] Brodovskaya A , Shiono S , Kapur J . Activation of the basal ganglia and indirect pathway neurons during frontal lobe seizures. Brain. 2021;144(7):2074–2091. 10.1093/brain/awab119 33730155 PMC8502464

[32] McCafferty C , Gruenbaum BF , Tung R , Li JJ , Zheng X , Salvino P , et al. Decreased but diverse activity of cortical and thalamic neurons in consciousness‐impairing rodent absence seizures. Nat Commun. 2023;14(1):117. 10.1038/s41467-022-35535-4 36627270 PMC9832004

[33] Wille C , Steinhoff BJ , Altenmüller DM , Staack AM , Bilic S , Nikkhah G , et al. Chronic high‐frequency deep‐brain stimulation in progressive myoclonic epilepsy in adulthood‐‐report of five cases. Epilepsia. 2011;52(3):489–496. 10.1111/j.1528-1167.2010.02884.x 21219312

[34] di Giacopo A , Baumann CR , Kurthen M , Capecchi F , Sürücü O , Imbach LL . Selective deep brain stimulation in the substantia nigra reduces myoclonus in progressive myoclonic epilepsy: a novel observation and short review of the literature. Epileptic Disord. 2019;21(3):283–288. 10.1684/epd.2019.1072 31225807

