# Supplementary material for: Restoring failed inhibition in the substantia nigra pars reticulata suppresses absence seizures in rats
Source: Epilepsia. 2025 Nov 3;67(2):966–78. doi: 10.1111/epi.18701 (PMC12927675; doi:10.1111/epi.18701)
Supplement: Supplementary file 1 — Appendix S1. [file EPI-67-966-s001.docx]

# Supplementary Material

**RESTORING FAILED INHIBITION IN THE SUBSTANTIA NIGRA PARS RETICULATA SUPPRESSES ABSENCE SEIZURES IN RATS**

Devin Palmer^1,2,^ and Patrick A. Forcelli^1,2,3^

**Supplementary Figure 1:** Putative dopaminergic unit activity within the SNr after SWD onset.

**Supplementary Figure 2:** SWD cycle phase-locking in GABA units.

**Supplementary Figure 3:** Closed-loop optogenetic inhibition of the SNr does not attenuate absence seizures in WAG/Rij rats.

**Supplementary Methods.**

**
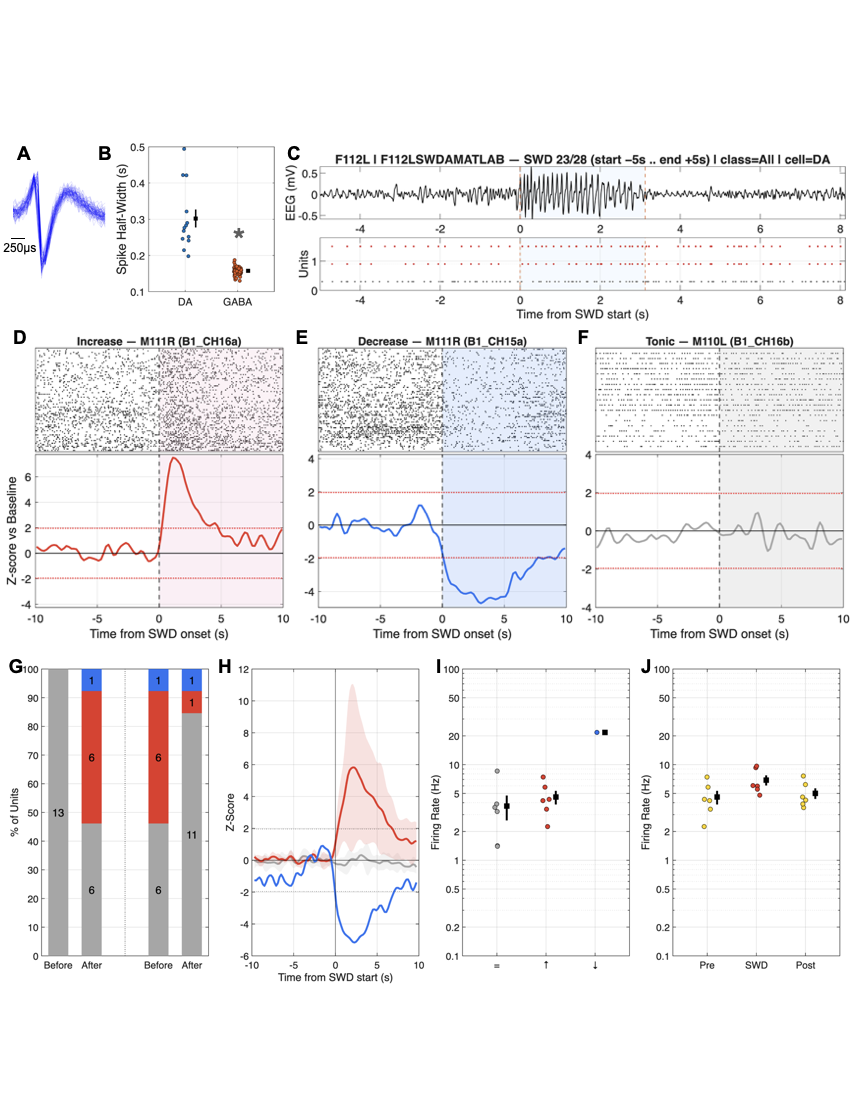
**

**Supplementary Figure 1. Single-unit dopaminergic activity within the SNr after SWD onset.** Of the 110 total units, only 13 showed waveform kinetics (spike half-width) consistent with DA neurons (**A**) Representative waveform from a putative dopaminergic neuron. **(B)** Peak/valley half-width of DA units was significantly longer than that of GABA units (Komogorov-Smirnov test, *p*=0.0001). **(C)** Expanded view of a single SWD time locked to raster plots for a phasic increase (red raster) and tonic unit (gray raster) (**D**) Raster plot (*top*) matched to the peri-event histogram of a unit showing tonic, (**E**) phasic increase and (**F**) phasic decrease preceding seizure start (seizure start = 0). Grey shaded region indicates tonic, pink shaded region indicates increase and blue shaded region indicates decrease after SWD onset. **(G)** DA unit activity pattern distribution before and after SWD start and end. Activity after SWD onset was heterogeneous. (**H**) Z-scored average activity in units that showed increased, decreased or tonic firing at SWD onset showed sustained activity throughout the SWD. **(I)** Baseline firing rates of DA units displaying tonic, increase, and decrease profiles. **(F)** For units that increased in activity we detected a significant effect of time bin (F_1.09,5.47_=18.35, p=0.006): firing rates were higher during the SWD periods compared with the PreSWD and PostSWD periods (ps<0.01, Holm-Sidak corrected).

**
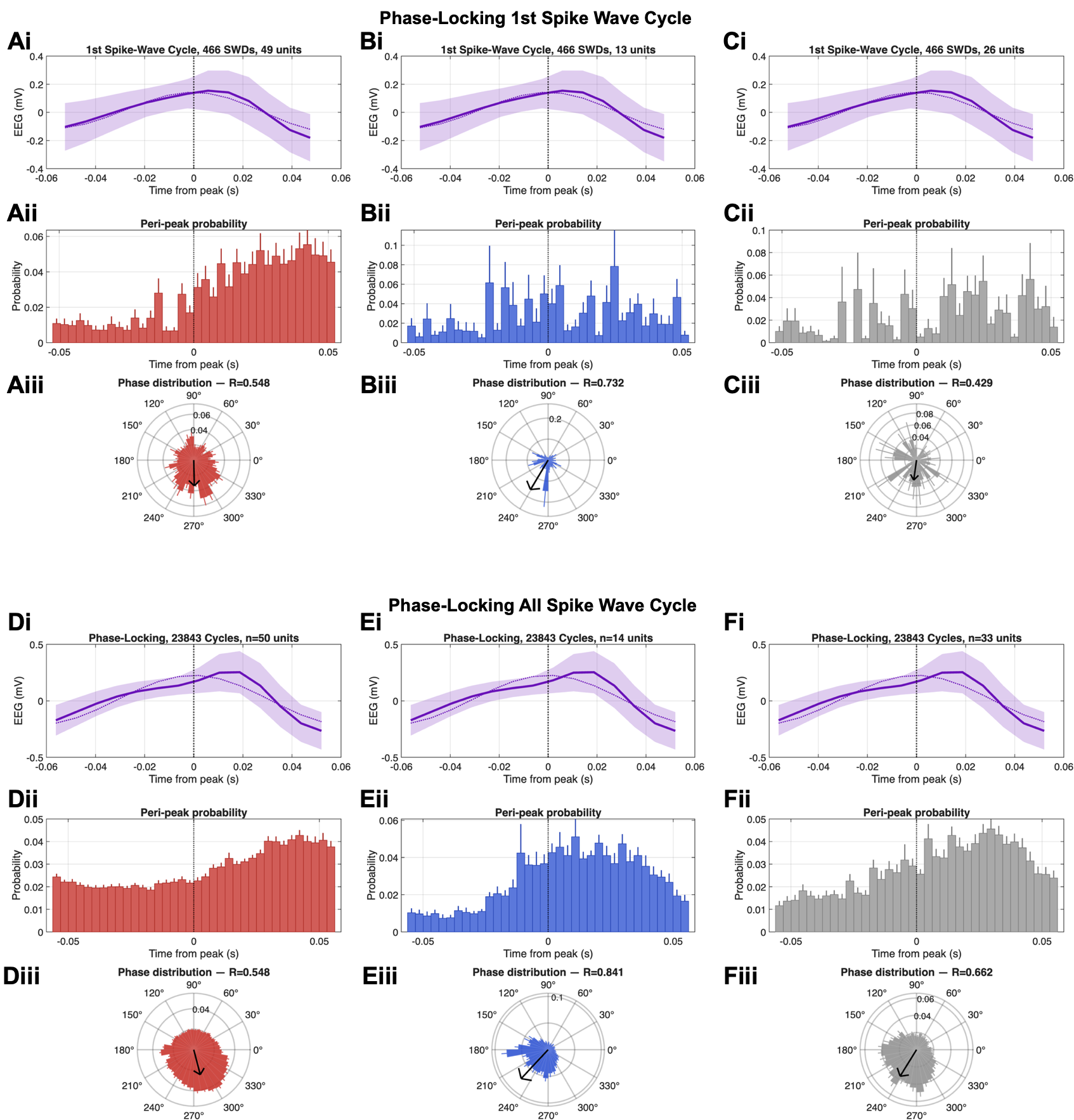
**

**Supplementary Figure 2. SWD cycle phase-locking in GABA units.** Phase-locking probabilities for each unit characterization were analyzed for phase preference in the initial spike wave cycle (A-C) and averaged across the entirety of SWD cycles (D-F). Units showing increased activation profiles at the start of SWDs showed increased firing probability in the positive phase of the 1^st^ SWD cycle (Ai-Aiii). A Rayleigh’s test for circular uniformity showed that the distribution of activity was not random, z(49)=14.74, mean angle = -88.66 degrees*, p*<0.001 during the 1^st^ phase of the SWD cycle (Aii-Aiii) and all SWD cycles, z(50)=15.01, mean angle = -75.37 degrees, *p*<0.001 (Dii-Diii). For units with decreased activation patterns a Rayleigh’s test for circular uniformity showed a non-random distribution of activity where units in the first phase of the SWD cycle (Bii-Biii) showed preference for the negative half of the phase (z(13)=6.99, mean angle = -120.99, *p* < 0.001) with similar preference throughout all spike wave cycles (Eii-Eiii) (z(14)=9.90, mean angle = -133.12, *p* < 0.001). Tonic units showed positive phase preference in the first SWD Cycle (Cii-Ciii) (z(26)=4.78, mean angle = -97.99, *p<*0.01), while showing a negative phase preference in all SWD cycles (Fii-Fiii) (z(33)=14.47, mean angle = -121.78, *p*<0.001).

**
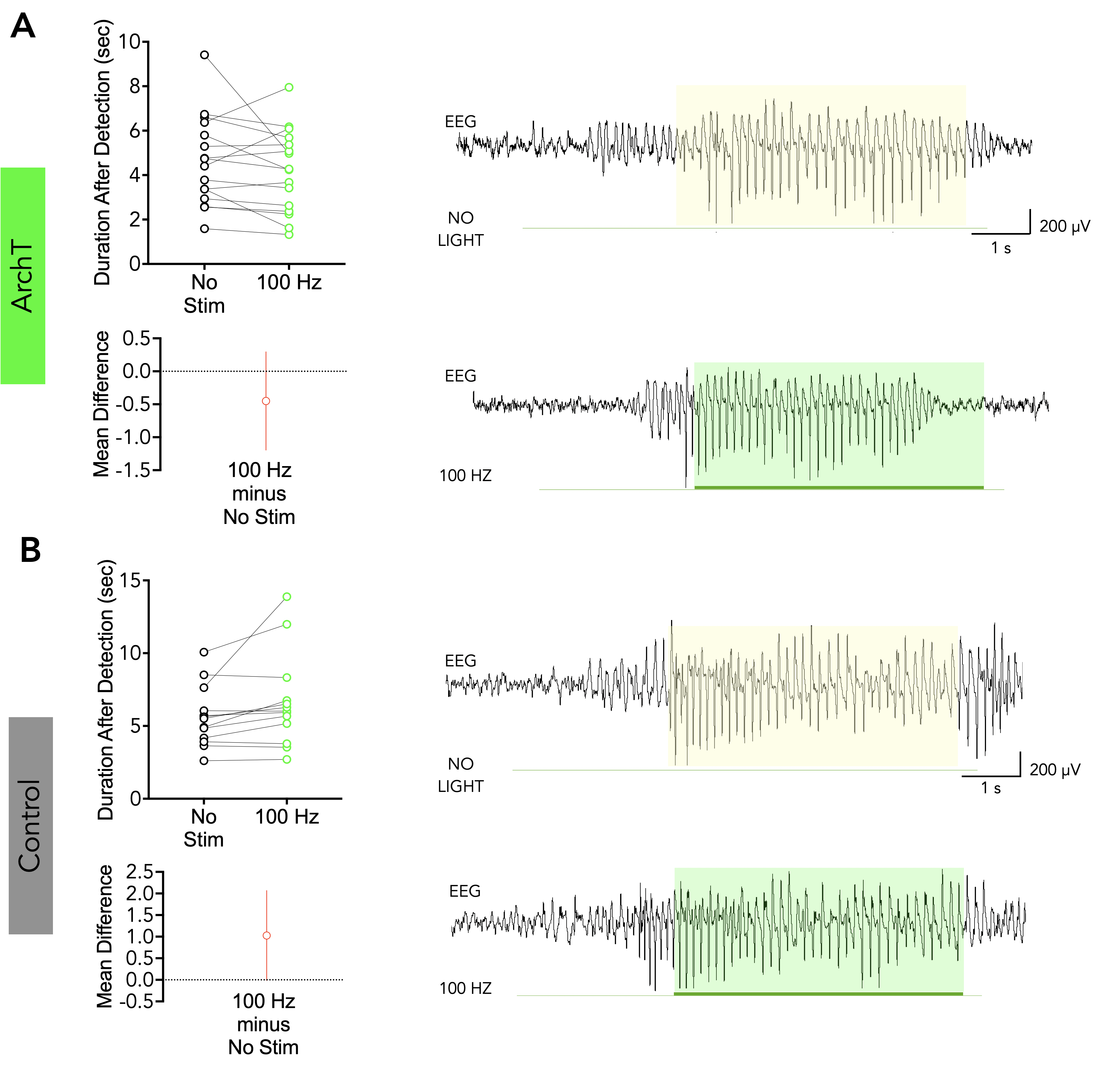
**

**Supplementary Figure 3. Closed-loop optogenetic inhibition of the SNr does not attenuate absence seizures in WAG/Rij rats. (A)** Optogenetic inhibition of the SNr in ArchT WAG/Rij rats did not affect the SWDs at 100 Hz light delivery (paired t-test, t=1.289, df=15, *p*=0.2170). Plots show individual replicates with mean and standard error. Mean differences (treated – control, with 95% confidence intervals) are plotted below each variable. Representative traces of a single SWD with no light (top/yellow) and 5 seconds of 100Hz 560nm light (bottom/green) **(B)** Optogenetic inhibition of the SNr in control WAG/Rij rats did not affect the SWDs at 100 Hz light delivery (*p =* 0.0529). Plots shosw individual replicates with mean and standard error. Mean differences (treated – control, with 95% confidence intervals) are plotted below each variable. As in A, representative traces of a single SWD with no light (top/yellow) and 5 seconds of 100Hz 560nm light (bottom/green).

**Supplementary Materials and Methods**

**Animals**

**General surgery and anesthesia**

Rats went under surgery for implantation of a 16-channel electrode array or bilateral virus injection, optic fiber and epidural EEG screw electrode placement. For surgery, rats were anesthetized (ketamine 75 mg/kg + dexmedetomidine 0.5 mg/kg, IP) and placed in a stereotaxic frame. Animals were allowed to recover for three weeks before recording. Fiber photometry animals were given an extra week of recovery to allow for proper viral expression.

**Microwire array implantation**

9 males and 8 female WAG/Rij rats were implanted with a 16-channel microwire array (35 μm tungsten electrodes with 175 μm spacing, Innovative Neurophysiology Inc.) in the SNr. Electrodes were implanted 5.5 mm posterior to bregma, 2.5 lateral to the midline, and 8.0 mm ventral to the dura with the head in a skull-flat orientation. A ground/reference electrode was wrapped around a stainless-steel screw and placed over the parietal cortex.

**Virus and optics: optogenetics**

Animals were randomized into either the active vector (ArchT) or control (GFP) condition. Animals received 1.0 μl of either rAAV8-CAG-ArchT-GFP (SNr inhibition; 9 males, 10 females) or rAAV8-CAG-GFP (control; 8 males, 5 females). Of these, 1 male and 1 female were dropped after analysis of histological analysis due to poor virus expression or off target fiber placement. The decision to drop animals was made while blind to both the genotype and the response to neurostimulation.

This resulted in final group sizes of 7 active vector males (open loop: n=7; closed loop: n=7); 8 control males (open loop: n=8, closed loop: n=8); 9 active vector females (open loop: n=9; closed loop: n=9); 5 control vector females (open loop: n=5; closed loop: n=4).

While the promotor for the ArchT construct is neuron-specific, it does not differentiate between excitatory neurons and inhibitory neurons. We previously showed equivalent effects of SNr inhibition using this strategy and a cre-dependent strategy that restricted expression to GAD expressing neurons.^4^ Epidural EEG screw electrode implantation and virus injection occurred as we have described.^4,7^ We implanted fiber optics bilaterally, and all animals were tested with bilateral manipulations, as prior studies have demonstrated that bilateral manipulation of basal ganglia is necessary for anti-seizure effects.^2^ Virus was injected bilaterally into the SNr (5.5 mm posterior to bregma, 2.5 lateral to the midline, and 8.0 mm ventral to the dura) with the head in a skull-flat orientation using a 30-gauge dental needle attached to a Hamilton syringe and syringe pump. Virus was sequentially injected into each SNr at a rate of 0.2 μl per minute. Following the injection, the needle was left in place for 7-10 min. Post injection, a fiber optic cannula (200 μm core, 0.22NA) was implanted 0.2 mm dorsal to each injection site (7.8 mm ventral to the dura). Prior to surgery, the efficiency of the fiber optics was measured to enable subsequent titration of laser power.

**Virus and optics: fiber-photometry**

Animals received 0.75μl bilateral injection (n=2 per animal) of pAAV1-iGABASnFR-WPRE-SV40 (3 males, 2 females). Surgical strategy, coordinates and recovery were the same as optogenetic experiments detailed above.

**EEG electrodes**

In the same surgical session, for optogenetic and fiber photometry experiments, epidural EEG screw electrodes were implanted bilaterally over the frontal lobe, parietal lobe, and two over the cerebellum (reference and ground). Epidural screw electrodes were routed into a plastic pedestal (InVivo One). Implants were secured in place using dental acrylic.^4,7^ The frontal electrodes were referenced to the cerebellum, the parietal electrodes were referenced to each other.

**Single unit data acquisition**

To isolate local field potential and seizure activity, a lowpass (30 Hz) filter was applied. To isolate unit activity, a bandpass (300 Hz to 6000 Hz) filter was applied. Data were digitally referenced to a common median reference in Offline Sorter. In the event that one or more channels displayed high levels of noise, they were dropped from analysis. An amplitude threshold (6 standard deviations of the mean signal amplitude) was applied to each channel to detect unit activity (32 sample per waveform). Units were sorted using k-mean clustering and the sorting for each unit was manually reviewed to remove artifacts. Only units with biologically plausible interspike intervals upon visual inspection of the auto-correlogram were included in the analysis (~8msec minimum interspike interval).

Spike train analysis was performed using a combination of in-house MATLAB scripts and NeuroExplorer (Plexon). An interval variable labeling each SWD was manually created for each file in Neuroexplorer. SWD onset was refined in MATLAB to lock to the peak of the first spike in a SWD on the EEG channel. A “PreSWD” interval was generated (10 seconds before SWD onset) and a “PostSWD” interval was generated (10 seconds after seizure end).

Baseline activity was defined as the 5 seconds preceding the onset of an SWD. Any SWD that started within 10 seconds of a preceeding SWD was excluded from further analysis.

Peri-event time histograms were generated based on the Z-transformed firing rate for each unit, with trials aligned by SWD onset and SWD offset with 0.2 second bins and a gaussian smoothing over nine bins. Units were classified by their response profile (increase, decrease, tonic). A unit was classified as an increase unit if its firing rate exceeded the 95% confidence intervals (Garwood estimation) for at least one bin.

To estimate the probability of a given unit increasing, decreasing, or displaying tonic firing in response to an SWD, we resampled the recordings on a unit-by-unit basis, for an equivalent number of intervals to the number of SWDs recorded during that session and repeated this resampling for 10,000 simulated sessions. Resampling was random but excluded sections of the recording within 20 of an SWD. Observed counts of response profiles were evaluated against the distribution from the resampled data.

To assess phase-locking of firing activity with SWDs, we down sampled the data, filtered the LFP data in the 6 to 11 frequency band to isolate SWDs, and analyzed firing probability as a function of SWD phase. We performed this analysis on a per unit level, as well as a per-animal level for units of each response profile type. Phase-firing coupling was analyzed statistically by Rayleigh’s test. We performed this analysis for the first spike-and-wave cycle of the SWD, first two cycles of the SWD, and for all SWD cycles extracted across all SWDs within an animal. In addition, we calculated the firing probability in the positive and negative phase of the SWD for each unit.

**Fiber photometry acquisition and analysis**

Acquisition was performed with a Neurophotometrics fiber photometry system (FP3001, Neurophotometrics LTD). iGABASnFR was excited at two wavelengths, 470nm and 415nm isosbestic control, by amplitude modulated signals from two light-emitting diodes reflected off dichroic mirrors and then coupled into a 2.5mm ceramic ferrule (400um core, 0.39NA, RWD Life Science Inc.). Emitted light was detected via a camera (FLIR BlackFly). Camera capture occurred at 60 FPS, evenly switching off between stimulation frequencies (30Hz per wavelength). Fluorescent signals from the camera were processed with Bonsai using the Neurophotometrics nodes.

Animals were tethered to a 3-channel EEG head stage preamplifier connected via a commutator (Pinnacle Technologies, Lawrence, KS) to the acquisition system (Pinnacle 8206). EEG was simultaneously acquired at 10 KHz with a 1 Hz hardware highpass filter in the head stage preamplifier (10x head stage gain) and amplified (50x). Electrographic activity was recorded using a PowerLab interface and LabChart 8 software (AD Instruments). Animals were recorded, unilaterally, for two hours. Recording of the other hemisphere occurred at least 24hours after the first recording.

1 male hemisphere and 1 female hemisphere were dropped from analysis due to lack of signal, verified by a lack of viral expression after histological analysis.

Using custom Matlab scripts, the EEG signal was down sampled and aligned to the raw photometry signal. Seizures were detected based on spike amplitudes, and seizure bins were labeled for subsequent analysis of iGABASnFR signal. Photometry signals (470nm and 415nm) were down sampled (30Hz). For each 2hr trial, data were detrended by regressing the isosbestic control signal (415nm) on the sensor signal (470nm) and then generating a predicted 415nm signal using the linear model generated during regression. The predicted 415nm channel was subtracted from the 475nm signal to remove movement, photo-bleaching, and fiber bending artifacts to obtain the ∆F/F signal which was time locked to the EEG signal for analysis.

**Optogenetic neuromodulation and EEG recording**

Fiber optics were coupled to a fiber-coupled diode-pulsed solid-state laser (560 nm for ArchT; Doric Lenses) by a fiber optic patch cord (fiber core 200 μm, NA 0.22). Laser power was calibrated with a power and energy meter to produce 10-12 mW out of the tip of the implanted fiber optic. Stimulation was controlled with LabChart software (AD Instruments) and a PowerLab interface.

We used two modulation techniques: Open-loop (continuous) and closed-loop (on-demand) neuromodulation. For open-loop experiments, 100Hz light was continuously applied for the entirety of the recording session. For closed-loop stimulation, seizures were detected in real time and were randomized to receive either stimulation or no stimulation, providing a within session control. The details for closed-loop neuromodulation follow precisely those we have previously described.^18,25^ In brief, we performed real time power transform on the EEG signal (5-15 Hz band) and tuned a threshold for each animal to ensure maximum seizure detection and minimize false positives. When stimulation was triggered, a 5 sec train of l00 Hz light pulses were delivered. We compared the efficacy of open-loop (i.e., continuous neuromodulation) to that of closed-loop stimulation on a within-subject basis.

We compared, between subjects, the effects of light delivery in active vector and control vector rats. Lack of effect of light delivery in opsin-negative (control vector) animals serves as a control for off-target effects, such as heating. Rats were tested on 4 sessions; the order of these sessions was pseudo-randomized and balanced across animals.

**EEG data analysis**

Recordings were scored offline in LabChart by an observer blinded to the animal genotype, optogenetic stimulation protocols, and frequency. SWDs were filtered (bandpass 1-50 Hz) and identified based on amplitude (peak to peak exceeding 2x the background activity, typically with a crescendo-decrescendo pattern to the amplitude). The onset of the SWD was defined as the first peak in the discharge.

**Histological verification**

Histological confirmation of virus injection and localization of fiber optic placement was conducted at the completion of all experiments by DP and reviewed by PAF. Histological confirmation was conducted while blinded to animal treatment and EEG results.
